# PRIMIS: design of a pivotal, randomized, phase 3 study evaluating the safety and efficacy of the nonsteroidal farnesoid X receptor agonist cilofexor in noncirrhotic patients with primary sclerosing cholangitis

**DOI:** 10.1186/s12876-023-02653-2

**Published:** 2023-03-15

**Authors:** Michael Trauner, Chuhan Chung, Kate Sterling, Xiangyu Liu, Xiaomin Lu, Jun Xu, Clare Tempany-Afdhal, Zachary D. Goodman, Martti Färkkilä, Atsushi Tanaka, Palak Trivedi, Kris V. Kowdley, Christopher L. Bowlus, Cynthia Levy, Robert P. Myers

**Affiliations:** 1 Division of Gastroenterology and Hepatology, Department of Medicine III, Medical University of Vienna, Währinger Gürtel 18-20, 1090 Vienna, Austria; 2 Gilead Sciences, Inc., 333 Lakeside Dr, Foster City, CA 94404 USA; 3 Department of Radiology, Ferenc Jolesz National Center for Image Guided Therapy, Brigham and Women’s Hospital, 75 Francis St, L1 Rm 050, Boston, MA 02115 USA; 4 Hepatic Pathology Consultation and Research, Inova Fairfax Hospital, 8110 Gatehouse Rd, Falls Church, VA 22042 USA; 5 Abdominal Center, Helsinki University Hospital, Helsinki, Finland; 6 Department of Medicine, Teikyo University School of Medicine, 2-11-1, Kaga, Itabashi-Ku, Tokyo, 173-8605 Japan; 7 National Institute for Health Research Birmingham Biomedical Research Centre, Centre for Liver and Gastrointestinal Research, Institute of Immunology and Immunotherapy, University of Birmingham, ITM Building, Mindelsohn Way, Edgbaston, Birmingham, B15 2TT UK; 8 Liver Institute Northwest, 3216 NE 45 Pl #212, Seattle, WA 98105 USA; 9 Division of Gastroenterology and Hepatology, UC Davis School of Medicine, 4150 V Street, Sacramento, CA 95817 USA; 10 Schiff Center for Liver Diseases, University of Miami, Jackson Medical Towers, 1500 NW 12 Ave, Suite 1101 ET, Miami, FL 33136 USA; 11 Inipharm, Bellevue, WA, USA; 12 OrsoBio, Inc., Palo Alto, CA, USA

**Keywords:** Primary sclerosing cholangitis, Farnesoid X receptor, Liver fibrosis

## Abstract

**Background:**

Primary sclerosing cholangitis (PSC) is a chronic progressive liver disease leading to biliary fibrosis and cirrhosis. Cilofexor is a nonsteroidal farnesoid X receptor agonist that demonstrated significant improvements in liver biochemistry and markers of cholestasis in patients with PSC in a phase 2 study. We describe here the rationale, design, and implementation of the phase 3 PRIMIS trial, the largest placebo-controlled trial in PSC.

**Methods:**

Adults with large-duct PSC without cirrhosis are randomized 2:1 to receive oral cilofexor 100 mg once daily or placebo for up to 96 weeks during the blinded phase. Patients completing the blinded phase are eligible to receive open-label cilofexor 100 mg daily for up to 96 weeks. The primary objective is to evaluate whether cilofexor reduces the risk of fibrosis progression compared with placebo. Liver biopsy is performed at screening and Week 96 of the blinded phase for histologic assessment of fibrosis. The primary endpoint—chosen in conjunction with guidance from the U.S. Food and Drug Administration—is the proportion of patients with ≥ 1-stage increase in fibrosis according to Ludwig histologic classification at week 96. Secondary objectives include evaluation of changes in liver biochemistry, serum bile acids, liver fibrosis assessed by noninvasive methods, health-related quality of life, and safety of cilofexor.

**Conclusion:**

The phase 3 PRIMIS study is the largest randomized, double-blind, placebo-controlled trial in PSC to date and will allow for robust evaluation of the efficacy and safety of cilofexor in noncirrhotic patients with large-duct PSC.

*Trial Registration:* ClinicalTrials.gov NCT03890120; registered 26/03/2019.

## Background

Primary sclerosing cholangitis (PSC) is a chronic and progressive cholestatic liver disease of unknown etiology characterized by persistent inflammation, stricturing, and obliterative fibrosis of the bile ducts, ultimately resulting in biliary cirrhosis [1–3]. Primary sclerosing cholangitis is an orphan disease with an estimated mean global prevalence of 10 per 100,000 population with ~ 30,000 individuals in the United States [4]. More than half of patients with PSC are men; affected individuals are generally diagnosed at ages 30–40 years, although pediatric and older ages at presentation also occur. Concomitant inflammatory bowel disease (IBD), most typically ulcerative colitis, occurs in the majority of patients [1, 5–7]. PSC is typically classified based on the location of biliary injury (small ducts, large ducts, or both) and the presence of underlying IBD. Patients with small-duct PSC have a more indolent course than those with classic large-duct PSC [6–8]. Although often asymptomatic in the early stages, as PSC progresses, patients can present with symptoms such as fatigue, abdominal pain, and pruritus, in addition to symptoms attributable to underlying IBD [7, 9–12]. With progression, potentially devastating complications such as dominant biliary strictures, recurring ascending cholangitis, and hepatic decompensation (eg, ascites, variceal hemorrhage, and hepatic encephalopathy [HE]) can occur [6–8]. Primary sclerosing cholangitis is also associated with metabolic bone disease and an increased risk of multiple malignancies, particularly cholangiocarcinoma, gallbladder cancer, and colorectal neoplasia [13, 14].

The clinical management of PSC is challenging. While multiple drugs have been evaluated including immunosuppressants, antibiotics, and antifibrotics, no pharmacologic therapy has been proven to improve clinical outcomes [3, 6, 7, 15]. Ursodeoxycholic acid (UDCA), a hydrophilic bile acid, improves liver biochemistry in most patients with PSC, but has not been shown to improve clinical outcomes, with high doses potentially harmful [7, 16, 17]. Liver transplantation is the only life-extending therapeutic option currently available, but the disease recurs in ~ 25% of patients at 5 years posttransplant [7, 18, 19]. The lack of effective treatment options, the clinical burden of PSC, and the variable, progressive nature of the disease have a substantial negative impact on health-related quality of life (HRQOL) and well-being, resulting in feelings of depression, anxiety, helplessness, and social isolation [12, 20–22].

A key pathophysiologic feature of PSC is dysregulation of bile acid homeostasis, characterized by altered bile acid composition and cholestasis [23, 24]. The farnesoid X receptor (FXR), a ligand-activated nuclear hormone receptor highly expressed in the liver, gallbladder, intestines, and kidney, is a key regulator of bile acid homeostasis [16, 25–27]. Activation of FXR by naturally occurring bile acids or synthetic FXR agonists, both within the intestine and hepatocytes, suppresses bile acid synthesis, inhibits bile acid uptake by hepatocytes, and promotes bile acid excretion, all of which lead to reduction of bile acid accumulation in the liver and enterohepatic circulation [16, 25, 26]. Farnesoid X receptor agonism also has anti-inflammatory effects, and may improve gut barrier function and reduce portal hypertension [16]. Activation of FXR, therefore, has pleiotropic effects that may be beneficial in cholestatic disorders such as PSC.

Cilofexor (formerly GS-9674) is a potent and selective nonsteroidal FXR agonist [27]. In preclinical models of liver fibrosis, cilofexor demonstrated anti-inflammatory and antifibrotic effects, reducing hepatic fibrosis and portal hypertension [28, 29]. To potentially improve tolerability and safety, cilofexor was designed and is dosed to predominantly activate intestinal FXR. This property is distinct from first-generation, bile acid–derived FXR agonists, such as obeticholic acid, which have greater effects on hepatic FXR and enterohepatic circulation [16, 27]. In a phase 2 study of 52 adults with large-duct PSC without cirrhosis, cilofexor 100 mg daily was well tolerated and had no adverse impact on IBD symptoms. Compared with placebo, cilofexor 100 mg significantly improved liver biochemistry, including serum alkaline phosphatase (ALP), γ-glutamyl transferase (GGT), alanine aminotransferase (ALT), and aspartate aminotransferase (AST), and reduced serum bile acids and serum C4 levels, a bile acid precursor. Cilofexor also demonstrated potential antifibrotic effects by reducing serum levels of the profibrogenic protein tissue inhibitor of metalloproteinase 1 [27].

Based on the results from the phase 2 study, the efficacy and safety of cilofexor is now under evaluation in the PRIMIS trial (ClinicalTrials.gov NCT03890120, registered 26/03/2019). This manuscript describes the design and rationale for the largest, multinational, randomized, double-blind, placebo-controlled, phase 3 study in patients with PSC to date.

## Methods

### Patients

Adult patients aged 18–75 years with a diagnosis of classic, large-duct PSC based on cholangiographic imaging (magnetic resonance cholangiopancreatography [MRCP], endoscopic retrograde cholangiopancreatography, or percutaneous transhepatic cholangiogram) are eligible for the study. Small duct PSC and other causes of liver disease including immunoglobulin G4 (IgG4)–related sclerosing cholangitis, autoimmune hepatitis/PSC overlap syndrome, and secondary sclerosing cholangitis are excluded. All patients have a liver biopsy at screening (or historical liver biopsy within 6 months of screening) that is deemed acceptable for interpretation by a central pathologist (Z.D.G.) and demonstrates stage F0–F3 fibrosis according to Ludwig classification [30]. Patients with clinical (eg, prior evidence of hepatic decompensation, platelet count < 150,000/mm^3^, and liver stiffness by vibration-controlled transient elastography [VCTE; FibroScan®, Echosens, Paris, France] > 20.0 kPa) and/or histologic evidence of cirrhosis are excluded from the study, because at the time of study initiation, the pharmacokinetics (PK) and tolerability of cilofexor in patients with cirrhosis were unclear. Patients are eligible regardless of baseline serum ALP concentration and use of UDCA, which must be stable for ≥ 6 months before screening. In patients with history of IBD, doses of biologic treatments, immunosuppressants, or systemic corticosteroids must be stable for ≥ 3 months prior to screening. Moderate to severe IBD—defined as a partial Mayo score > 4 and/or a score in the screening visit rectal bleeding domain > 1 (unless bleeding is due to perianal disease)—is exclusionary. Additional key inclusion and exclusion criteria are listed in Table 1.

**Table 1:** PRIMIS key inclusion and exclusion criteria

Key inclusion criteria	Key exclusion criteria
Men and nonpregnant, nonlactating women aged 18–75 years with diagnosis of large-duct PSC based on cholangiogram Liver biopsy at screening deemed acceptable for interpretation and demonstrates stage F0–F3 fibrosis in opinion of central reader Historical liver biopsy within 6 months of screening visit may be accepted if deemed acceptable for interpretation Individual has the following laboratory parameters at screening visit as determined by central laboratory: Platelet count ≥ 150,000/mm^3^ eGFR ≥ 30 mL/min (per Cockcroft-Gault) ALT ≤ 8 × ULN Total bilirubin < 2 mg/dL unless individual has Gilbert's syndrome or hemolytic anemia INR ≤ 1.4 unless due to therapeutic anticoagulation Negative antimitochondrial antibody For patients on UDCA, dose of UDCA must be stable for ≥ 6 months before screening; for those not on UDCA, no UDCA use for ≥ 6 months before screening For patients on biologics, immunosuppressants, or systemic corticosteroids, dose must be stable for ≥ 3 months before screening and remain stable throughout trial	Current or prior history of any of following: Cirrhosis defined by stage F4 fibrosis according to Ludwig classification or equivalent on liver biopsy; decompensated liver disease including ascites, HE, and variceal hemorrhage; or liver stiffness > 20.0 kPa by FibroScan Liver transplantation Cholangiocarcinoma or HCC Ascending cholangitis within 30 days of screening Presence of percutaneous drain or biliary stent Other causes of liver disease History of malignancy within 5 years of screening^a^; unstable cardiovascular disease; hypercoagulable condition, or venous or arterial thromboembolic disease; or intestinal resection or malabsorptive condition CP score > 6 unless due to alternate etiology MELD score > 12 unless due to alternate etiology HIV, HBV, or HCV infection Current moderate–IBD, including ulcerative colitis, Crohn's disease, and indeterminate colitis

^a^Except adequately treated carcinoma in situ of cervix, basal or squamous cell cancer, or other localized nonmelanoma skin cancer

*ALT* alanine aminotransferase; *CP* child–pugh; *eGFR* estimated glomerular filtration rate; *HCC* hepatocellular carcinoma; *HBV* hepatitis B virus; *HCC* hepatocellular carcinoma; *HCV* hepatitis C virus; *HE* hepatic encephalopathy; *HIV* human immunodeficiency virus; *IBD* inflammatory bowel disease; *INR* international normalized ratio; *MELD* model for end-stage liver disease; *PSC* primary sclerosing cholangitis; *UDCA* ursodeoxycholic acid; *ULN* upper limit of normal

### Study design

**Fig. 1:**
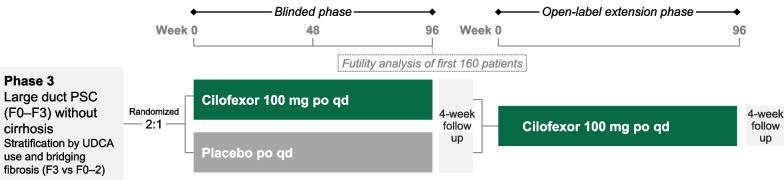
PRIMIS study design. PSC, primary sclerosing cholangitis; UDCA, ursodeoxycholic acid

This phase 3 study consists of 2 phases: a blinded phase and an open-label extension (OLE) phase (Fig. 1). The blinded phase includes a 10-week screening period, 96 weeks of treatment, and a follow-up visit 4 weeks after completion of Week 96 or early termination (ET). Owing to the rarity and high unmet need of PSC, and the requirement for placebo treatment of nearly 2 years in some patients, eligible patients are randomized unevenly in a 2:1 ratio to receive active treatment with oral cilofexor 100 mg or placebo daily. Randomization is stratified by presence or absence of UDCA use and bridging fibrosis (Ludwig fibrosis stage F3 vs. F0–F2) on screening liver biopsy, the latter reflecting an important prognostic factor in this population [15]. After screening, in-clinic study visits occur at baseline, Weeks 4, 8, 12, 24, 36, 48, 60, 72, 84, and 96 or ET, and the follow-up visit. This 96-week blinded phase will provide evidence for the safety and efficacy of cilofexor vs placebo in different patient subgroups at risk for progressive hepatic fibrosis and the development of cirrhosis.

Patients who do not permanently discontinue study drug and complete the blinded phase Week 96 or follow-up visit are eligible to enter the OLE phase, and receive open-label cilofexor 100 mg daily for up to 96 weeks. The OLE phase includes a 4-week rollover period, 96 weeks of treatment, and a follow-up visit 4 weeks after completion of OLE Week 96 or ET. After rollover, in-clinic study visits occur at OLE baseline, Weeks 4, 24, 48, 72, and 96 or ET, and the follow-up visit. This 96-week OLE phase will provide additional data regarding the long-term safety and efficacy of cilofexor.

The study was initiated in March 2019 with an initial focus on activation of North American study sites. In autumn 2019, changes to the protocol were made with an amendment based on patient and investigator requests to decrease study burden and reduce recruitment delays. These changes included increasing the upper age cutoff from 70 to 75 years, extension of the screening window from 8 to 10 weeks, addition of a 14-day visit window for the initial MRCP, and an optional liver stiffness measurement by VCTE. Due to the Covid-19 pandemic, and requests by sites and potential subjects to facilitate enrollment, an additional amendment was initiated in the spring of 2020 that included reductions in the numbers of on-site study visits, collections of samples for biomarkers, and PSC-related HRQOL questionnaires. A fourth amendment in June 2021 added an interim futility analysis based on the primary endpoint after the first 160 randomized and dosed patients completed the blinded phase of the study and the OLE phase, based on requests of patients and investigators.

### Dosing rationale

The oral 100 mg daily dosage of cilofexor was selected for the phase 3 study based on safety, efficacy, and PK and pharmacodynamic data from the dose-ranging phase 2 PSC study [27], data from a phase 2 study in nonalcoholic steatohepatitis [31], and phase 1 studies in healthy subjects [32, 33]. In the phase 2 PSC study, improvements in liver biochemistry, including serum ALP, ALT, AST, and GGT, and serum bile acids after 12 weeks of treatment with cilofexor were greater with the 100 versus 30 mg dose, while the incidence of treatment-emergent adverse events (AEs), including Grade 2 or 3 pruritus, was mostly similar between the two doses [27]. In the phase 1 studies, cilofexor exhibited less than dose-proportional increases in systemic exposure at doses > 100 mg [32, 33], suggesting that higher doses may provide only marginal incremental benefits in efficacy, with a higher potential risk of AEs including pruritus. The combined safety, efficacy, and PK data supported evaluation of cilofexor 100 mg daily in patients with PSC in the present phase 3 study.

### Study objectives and endpoint rationale

Because of the heterogeneity and slow progression of PSC, Level-1 clinical endpoints measuring outcomes of death, liver transplantation, and cholangiocarcinoma are challenging due to the low incidence of clinical events in phase 2 and 3 studies of typical size and duration [34, 35]. To overcome this challenge, the International PSC Study Group (IPSCSG) has proposed several biomarkers as surrogate endpoints, including serum ALP, a well-established biomarker of cholestasis [36]. For a biomarker to be accepted as a surrogate endpoint, it must be “reasonably likely to predict clinical benefit” [35, 36]. Although ALP is a useful and appropriate endpoint for small, short-term, phase 2 studies, recent pooled data from the simtuzumab phase 2 PSC trial demonstrated large inter- and intraindividual variations in ALP activity and a lack of association between changes in ALP alone with disease progression [37]. Currently, none of the potential endpoints proposed by IPSCSG is approved by global regulatory authorities for use as a surrogate to support accelerated approval of experimental drugs in PSC [35, 36].

**Fig. 2:**
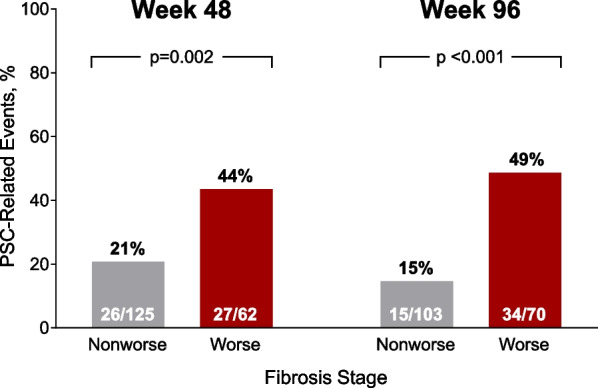
Nonworsening of fibrosis at Weeks 48 and 96 among noncirrhotic patients in the phase 2 simtuzumab primary sclerosing cholangitis (PSC) study was associated with significantly reduced rate of PSC-related events. *p* values by Fisher’s exact test

**Fig. 3:**
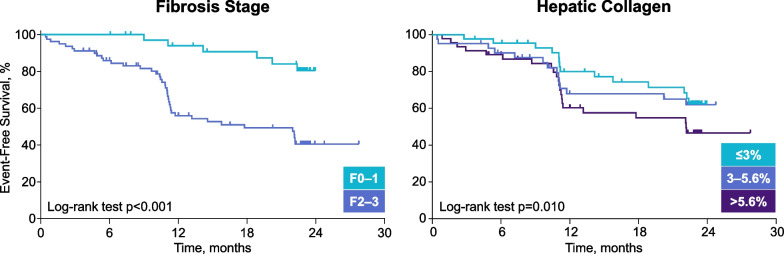
Greater fibrosis burden at baseline, as defined by Ludwig fibrosis stage or hepatic collagen content by morphometry, was associated with a significantly increased risk of disease progression among noncirrhotic patients with PSC in the phase 2 simtuzumab study. Disease progression was defined by progression to cirrhosis (F4), ascending cholangitis, hepatic decompensation, liver transplantation, or death. [15] *p* values by log-rank test

The primary objective of the PRIMIS trial is to evaluate whether cilofexor reduces the risk of fibrosis progression among noncirrhotic patients with large-duct PSC. As such, the primary endpoint is the proportion of patients with liver fibrosis progression, defined as ≥ 1-stage increase in fibrosis according to Ludwig classification (stage 1, cholangitis/portal hepatitis; stage 2, periportal fibrosis; stage 3, septal fibrosis or bridging necrosis; stage 4, biliary cirrhosis) from baseline to Week 96. The Ludwig staging system has previously shown strong associations with the occurrence of liver-related events (eg, ascites, HE, and transplantation), including in an international cohort study [35, 38]. In the simtuzumab study, no worsening of fibrosis was associated with a significantly reduced risk of clinical events at Weeks 48 and 96 among noncirrhotic patients with PSC (Fig. 2) [15]. In addition, higher levels of fibrosis content at baseline, based on either traditional Ludwig staging or a continuous score, such as hepatic collagen by morphometry, were associated with clinical events (Fig. 3). Based on this prognostic relevance, a histology-based endpoint is considered by the U.S. Food and Drug Administration (FDA) to be an acceptable surrogate endpoint in PRIMIS to support the accelerated approval of cilofexor for the treatment of PSC. While draft guidance from the European Medicines Agency has recommended use of the Nakanuma staging system [38], this system has similar reproducibility and prognostic value to Ludwig classification, despite the inclusion of more histologic parameters and requirement for orcein staining, which is not routinely performed.

The ultimate goal for any therapy in PSC is to prevent liver-related complications. While the PRIMIS trial is underpowered to demonstrate a benefit of cilofexor on clinical events, data on relevant complications are being prospectively recorded and adjudicated in a blinded fashion according to standardized criteria by a committee of experts. Specifically, a composite of clinical events that constitute this clinical efficacy endpoint have been defined and include: (1) progression to cirrhosis (liver biopsy showing F4 fibrosis according to Ludwig classification in the opinion of the central reader or clinical evidence of cirrhosis); (2) events of hepatic decompensation (clinically apparent ascites, Grade ≥ 2 HE by West Haven criteria requiring treatment, and portal hypertension-related upper gastrointestinal bleeding identified by endoscopy and requiring hospitalization); (3) liver transplantation or qualification for liver transplantation (Model for End-Stage Liver Disease [MELD] score ≥ 15 on ≥ 2 consecutive occasions ≥ 4 weeks apart); and (4) all-cause mortality. The hepatic events adjudication committee will also review all cases of cholangiocarcinoma, hepatocellular carcinoma, ascending cholangitis, and dominant strictures as events of special interest.

The secondary objectives of the PRIMIS trial include assessment of the safety and tolerability of cilofexor, and evaluation of changes in liver biochemistry, serum bile acids, liver fibrosis assessed by histology (specifically fibrosis improvement) and noninvasive methods (eg, Enhanced Liver Fibrosis [ELF™] score [Siemens Healthineers, Erlangen, Germany], and liver stiffness by VCTE), and HRQOL. Biochemical responses and noninvasive markers of fibrosis may support the beneficial effects of cilofexor on disease progression to supplement the primary histologic endpoint and prove useful for prognostication and disease monitoring in the future. Indeed, baseline levels and changes in these fibrosis markers were associated with risk of disease progression among patients in the simtuzumab trial [15]. A key secondary endpoint to be evaluated in PRIMIS is the proportion of patients with ≥ 25% relative reduction in serum ALP concentration from baseline (biochemical response) and no worsening of fibrosis according to Ludwig classification (histologic response) at Week 96. This endpoint is consistent with draft guidance from the European Medicines Agency and recommendations from IPSCSG.

Among a variety of HRQOL measures to be collected in the PRIMIS trial is the disease-specific PSC-patient-reported outcome (PSC-PRO) instrument, which was developed according to FDA guidelines to complement clinical outcomes data. In a preliminary validation of PSC-PRO in patients with PSC [12], it demonstrated excellent internal consistency, discriminant validity, and test–retest reliability, and specific domains within the instrument were well correlated with relevant domains of other HRQOL instruments including the 36-Item Short Form Health Survey, Chronic Liver Disease Questionnaire, and PBC-40. Importantly, PSC-PRO could differentiate patients with PSC according to presence and severity of cirrhosis. The primary, secondary, and exploratory endpoints of PRIMIS are listed in Table 2.

**Table 2:** Study endpoints

Primary endpoint
Proportion of patients with liver fibrosis progression defined as ≥ 1-stage increase in fibrosis according to Ludwig classification at Week 96 (blinded phase)
Secondary endpoints (at Week 96 [blinded phase])
Changes from baseline in serum concentrations of ALP, GGT, ALT, and bile acids
Proportion of patients with ≥ 25% relative reduction in serum ALP concentration from baseline (biochemical response) and no worsening of fibrosis according to Ludwig classification (histologic response)
Changes from baseline in liver fibrosis, including hepatic collagen content, fibrosis improvement, progression to cirrhosis (according to Ludwig classification), and noninvasive markers of fibrosis, including liver stiffness by FibroScan and ELF score
Changes from baseline in HRQOL based on disease-specific PSC-PRO
Exploratory endpoints
Changes from baseline in markers of liver injury and function, including bilirubin, albumin, and INR
Changes in hepatitis and cholangitis activity, and bile duct loss (according to Nakanuma classification) at Week 96 (blinded phase)
Changes from baseline in biliary stricture severity as measured by MRCP at Week 96 (blinded phase)
Changes from baseline in HRQOL measures and health resource utilization
Changes from baseline in Mayo risk score and Amsterdam-Oxford score
Incidence of PSC-related complications including hepatic decompensation, ascending cholangitis, dominant strictures, cholangiocarcinoma, HCC, liver transplantation or meeting minimal listing criteria for transplantation (ie, MELD score ≥ 15), and mortality
Event-free survival, defined as time to first clinical event. including histologic or clinical progression to cirrhosis, hepatic decompensation, liver transplantation, and all-cause mortality or last follow-up, whichever occurs first

*ALP* alkaline phosphatase; *ALT* alanine aminotransferase; *ELF* enhanced liver fibrosis score; *GGT* γ-glutamyltransferase; *HCC* hepatocellular carcinoma; *HRQOL* health-related quality of life; *INR* international normalized ratio; *MELD* model for end-stage liver disease; *MRCP* magnetic resonance cholangiopancreatography; *PSC* primary sclerosing cholangitis; *PSC-PRO* PSC-patient-reported outcome

### *Study assessments*

Liver biopsy for histologic staging of fibrosis is performed at screening and Week 96 during the blinded phase and reviewed by the central reader. Biopsies are performed under ultrasound guidance, when possible, to reduce the risk of AEs. Measurements of serum markers of liver injury and function, including ALP, GGT, ALT, AST, bilirubin, albumin, and INR, and clinical liver assessments, including ascites, HE, and calculation of MELD and Child–Pugh scores, are performed at screening and all in-clinic study visits during the blinded and OLE phases. Serum bile acids, noninvasive markers of fibrosis, and health resource utilization and HRQOL questionnaires are evaluated at specified time points (Table 3). Imaging of biliary and pancreatic ducts by MRCP is performed at baseline, and Weeks 48 and 96 or ET during the blinded phase, read locally for any clinically significant abnormalities to ensure patient safety, and reviewed by a central reader (C.T.A.) to evaluate changes to the biliary tree according to standardized criteria [39].

**Table 3:** Study assessments

Parameter	Assessment	Timing
Histologic staging of fibrosis	Liver biopsy	Screening and Week 96 (or ET) during blinded phase
Liver biochemistry	ALP, GGT, ALT, AST, bilirubin, albumin, and INR	Screening, baseline, and Weeks 4, 8, 12, 24, 36, 48, 60, 72, 84, and 96 (or ET) during blinded phase; OLE baseline, and Weeks 4, 24, 48, 72, and 96 (or ET)
Clinical liver assessments	Ascites, hepatic encephalopathy, calculation of MELD and CP scores	
Markers of bile acid homeostasis	Serum bile acids	Screening, baseline, and Weeks 4, 12, 24, 48, 72, and 96 (or ET) during blinded phase; OLE baseline, and Weeks 48 and 96 (or ET)
Noninvasive markers of liver fibrosis	Liver stiffness by FibroScan	Screening and every 24 weeks (or ET) during blinded phase; OLE baseline and every 24 weeks (or ET)
	ELF	Screening, baseline, and Weeks 4, 12, 24, 48, 72, and 96 (or ET) during blinded phase; OLE baseline, and Weeks 48 and 96 (or ET)
Biliary strictures	MRCP	Baseline, and Weeks 48 and 96 (or ET) during blinded phase
Health resource utilization and HRQOL	SIBDQ (for patients with history of IBD), CLDQ, EQ-5D,^a^ and PSC-PRO	Baseline and every 24 weeks (or ET) during blinded phase; OLE baseline and every 24 weeks (or ET)
Safety	AEs, clinical laboratory tests, vital sign assessments, and concomitant medications	Various time points throughout study

^a^EuroQol, Rotterdam, the Netherlands

*AEs* adverse events; *ALP* alkaline phosphatase; *ALT* alanine aminotransferase; *AST* aspartate aminotransferase; *CLDQ* chronic liver disease questionnaire; *CP* child–pugh; *ELF* enhanced liver fibrosis score; *ET* early termination; *GGT* γ-glutamyltransferase; *HRQOL* health-related quality of life; *IBD* inflammatory bowel disease; *INR* international normalized ratio; *MELD* model for end-stage liver disease; *MRCP* magnetic resonance cholangiopancreatography; *OLE* open-label extension; *PSC-PRO* primary sclerosing cholangitis-patient-reported outcome; *SIBDQ* short inflammatory bowel disease questionnaire

Safety is assessed through the reporting of AEs, clinical laboratory tests including lipid profiles, and vital sign assessments at various time points throughout the study; concomitant medication usage is also assessed (Table 3). An independent, external Data and Safety Monitoring Board (DSMB) comprising 2 hepatologists and a PhD statistician convenes after 50 patients have completed the Week 4 visit, and approximately every 6 months thereafter during the blinded and OLE phases to monitor the study for safety events. To mitigate the potential risk of liver injury, patients are monitored closely with defined rules for study drug withholding due to elevated liver tests; all potential cases of drug hepatotoxicity are adjudicated by an independent drug-induced liver injury adjudication committee. For patients with new or worsening pruritus, management strategies include nonpharmacologic and pharmacologic interventions (eg, topical corticosteroids, oral antihistamines, and bile acid sequestrants), and treatment interruption or dose reduction of cilofexor (to 30 mg daily) in cases of intolerable pruritus. At the time of writing of this manuscript, there have been four independent DSMB assessments of the data with the conclusion that the study should proceed without change to the protocol.

### Statistical analyses

Considerable efforts were undertaken to balance the needs for a statistically rigorous study while limiting the exposure of patients with PSC to long-term treatment with placebo. As such, eligible patients were randomized 2:1 to receive cilofexor (n = 267) or placebo (n = 133). This sample size of 400 patients in total was calculated to have > 80% power to detect an absolute difference of 15% in the percentages of patients who meet the primary histologic endpoint at blinded study phase Week 96. The power analysis was conducted using Pearson’s chi-square test at a 2-sided significance level of 0.05. This calculation assumed that 25% of patients would discontinue the study prematurely (considered as treatment failures) and that among those with nonmissing response data at blinded phase Week 96, 20% in the cilofexor arm and 40% in the placebo arm would meet the primary endpoint. The 40% estimate of fibrosis progression in the placebo arm is based on Week 96 data from patients without cirrhosis in the simtuzumab trial [15].

The PRIMIS trial contains 3 planned analyses. First, an interim futility analysis based on the primary histologic endpoint will be conducted after the first 160 randomized and dosed patients have completed Week 96 or ET assessments in the blinded phase. A predictive power approach, which calculates the probability of observing a statistically significant result for the primary endpoint given the interim data, will be used for this assessment. Specifically, the data monitoring committee may recommend early termination of the study due to futility if the predictive power is ≤ 10%. Second, the primary analyses on the primary, secondary, and exploratory endpoints will be conducted after all randomized and dosed patients have completed Week 96 or ET assessments in the blinded phase. The final analyses will be performed after all patients have completed the OLE phase follow-up visit.

For the primary analysis, a stratified Mantel–Haenszel test will be used to compare the difference in proportions of patients with liver fibrosis progression at blinded phase Week 96 (primary endpoint) between cilofexor and placebo at a 1-sided significance level of 0.025, adjusting for baseline UDCA use and fibrosis stage (Ludwig fibrosis score F3 vs. F0–F2) on screening liver biopsy. Patients with missing data on liver fibrosis or with clinical events occurring prior to Week 96 will be analyzed as treatment failures. Secondary efficacy endpoints will be tested sequentially in a prespecified order after the primary endpoint has been met. If a 1-sided *p* value ≤ 0.025 is achieved for one endpoint, the next endpoint will be evaluated; otherwise, testing of the remaining endpoints will cease. For these analyses, an analysis of covariance model will be used for continuous and ordinal outcomes, and a stratified Mantel–Haenszel test will be used for binary outcomes, adjusting for baseline stratification factors. Exploratory endpoints and safety will be summarized using descriptive methods by treatment group.

## Discussion

The PRIMIS trial is the largest multinational, randomized, double-blind, placebo-controlled phase 3 study in PSC to date. Screening for the study initiated in March 2019, and by December 2020, 204 sites in 16 countries in Australasia, Europe, Japan, and North America have been activated. An additional site was later activated. In all, 419 noncirrhotic patients with large-duct PSC have been enrolled and 416 were dosed. The screen failure rate was 29% (171/590 patients screened), with major reasons for screen failure including laboratory abnormalities, current or recent history of PSC-related complications, and noneligible screening liver biopsy, each occurring in > 10% of screen-failed subjects. Among patients who were dosed and have available baseline data as of February 14, 2022, median age is 44 years (range 18–74), 62% are men, 70% have history of IBD, 59% are on UDCA, and 25% have F3 fibrosis on screening liver biopsy. Median serum ALP concentration is 173 U/L (interquartile range 107, 292), with 69% of enrolled patients having elevated serum ALP.

The primary endpoint of the PRIMIS trial will provide clinically relevant information on whether cilofexor can reduce progression of liver fibrosis in noncirrhotic patients with large-duct PSC. Currently, no approved pharmacologic treatment for PSC exists, in part, due to lack of clarity regarding which endpoints may reliably serve as surrogates of clinical outcomes in PSC. The rarity of PSC and its generally prolonged natural history pose inherent difficulties in performing adequately powered, clinical outcomes studies [35, 36]. For example, in a large population-based study from the Netherlands, Boonstra and colleagues [5] reported an estimated median survival from diagnosis to transplant or PSC-related death of 21 years. In a large, 5-year trial of high-dose UDCA, the incidence of clinical events including liver transplantation and death was ~ 3% annually in patients receiving placebo [40]. As patients with cirrhosis are excluded from PRIMIS, a lower rate of clinical events would be expected in this trial. Considering these challenges, fibrosis progression on liver biopsy was chosen, in close collaboration with the FDA, as the primary endpoint of PRIMIS and endorsed by the FDA as acceptable for accelerated approval of cilofexor assuming success of the phase 3 study. Data on progression to cirrhosis—defined histologically or clinically, adjudicated centrally, and expected to occur primarily in patients with bridging (F3) fibrosis at baseline (25% of patients in PRIMIS)—will contribute to the evidence regarding the efficacy of cilofexor.

The strengths and limitations of the study reflect the inherent challenge of conducting a study in a chronic disease indication such as PSC with variable clinical course. Regulatory support of the histologic endpoints to be evaluated in the PRIMIS trial is based on the well-accepted observation that severity of liver fibrosis correlates with risk of clinical events in PSC, as well as other liver diseases [15]. A major shortcoming of liver histology in PSC is sampling variability due to the patchy distribution of injury and fibrosis. Dominant strictures may affect only one hepatic lobe or segment, which may be missed by random sampling of liver tissue (typically percutaneously from the right lobe) and the inability to obtain serial biopsies from the same location. The invasive nature and potential complications of liver biopsy are additional limitations. For this reason, the clinical practice guidelines of the American Association for the Study of Liver Diseases and European Association for the Study of the Liver do not currently recommend liver biopsy in the *routine* management of PSC, except to rule out other diagnoses or diagnose small-duct PSC [2, 41]. A requirement for serial liver biopsies may also impede patient recruitment in trials of novel therapies for PSC. However, this was not a major obstacle to enrollment in PRIMIS, confirming findings from the simtuzumab study, which required three liver biopsies per patient over 96 weeks. Thus, the inherent limitations of a biopsy-based primary endpoint in PRIMIS were governed by the need to conduct a regulatory-defined endpoint within a reasonable time frame.

The phase 2 study of cilofexor in noncirrhotic patients with PSC demonstrated that cilofexor significantly improved markers of cholestasis, liver biochemistry, and bile acid homeostasis [27]. Several key secondary endpoints in the PRIMIS trial include changes in liver biochemistry and noninvasive markers of fibrosis, such as liver stiffness by VCTE and ELF score. These markers were significantly associated with PSC-related complications in multiple prior studies [15, 42, 43]. For example, in the simtuzumab study among patients without cirrhosis, those with baseline liver stiffness by VCTE ≥ 8.2 kPa had an ~ sevenfold risk of disease progression. After adjusting for baseline values, an increase of 1 kPa during follow-up was associated with an 8% increase in risk of events (hazard ratio [HR] per 1 kPa: 1.08; 95% confidence interval [CI] 1.03, 1.13). Similarly, greater ELF score at baseline (HR per 0.5 units: 1.34; 95% CI 1.21, 1.49) and greater increases over time (HR per 0.5 units: 1.36; 95% CI 1.17, 1.59) were associated with higher likelihood of disease progression. Data in the larger population of PRIMIS will inform whether cilofexor improves these markers over the longer term and supplement data obtained from liver histology regarding the putative antifibrotic effects of this therapy. Moreover, the systematic, protocol-based evaluation of liver fibrosis based on histology and noninvasive markers at baseline and over time will provide valuable information regarding the natural history of noncirrhotic large-duct PSC, and help confirm the utility of these surrogate markers for risk stratification and disease monitoring.

The long-term safety profile of cilofexor in PSC has been evaluated in the 96-week OLE of the phase 2 study and will be further evaluated in the PRIMIS trial. Available data from the phase 1 and 2 studies in healthy subjects, and patients with PSC and nonalcoholic steatohepatitis indicate that cilofexor was generally well tolerated [27, 31–33, 44]. Pruritus, a symptom commonly associated with cholestatic liver diseases [7, 9–11] and a known complication of FXR agonist therapy [17], was lower in patients treated with cilofexor vs placebo during the 12-week double-blind period of the phase 2 PSC study. Premature discontinuation of therapy was observed in 1 patient (2%) treated with cilofexor during the double-blind phase [27] and in 5 (11%) during the OLE phase of that study. Given the important impact of pruritus on HRQOL in patients with PSC, a protocol-defined pruritus management plan was implemented in PRIMIS to potentially mitigate the risk of treatment discontinuations due to this symptom. This plan includes temporary interruption of study drug dosing, a dose-escalation scheme at defined study intervals (beginning at cilofexor 30 mg or placebo), and supportive management with antipruritic medications.

## Conclusion

The PRIMIS trial is a pivotal phase 3 study designed to evaluate the long-term effects of cilofexor on fibrosis progression, liver biochemistry, and HRQOL, as well as PSC-related complications, over a 192-week treatment period in noncirrhotic patients with large-duct PSC. This study will provide valuable data on the natural history of PSC, with histologic and serologic assessments in patients receiving placebo over the 96-week blinded phase. This largest randomized controlled PSC study will allow for robust investigation of the long-term therapeutic efficacy and safety of cilofexor and should provide critical insights on the disease state and natural progression of noncirrhotic large-duct PSC.

## Data Availability

The datasets generated during the current study are not publicly available because the study described is currently in-progress. Once the study is completed, the data are available from the study sponsor on reasonable request. Data requests should be sent to datarequest@gilead.com.

## Abbreviations

AE: Adverse event
ALP: Alkaline phosphatase
ALT: Alanine aminotransferase
AST: Aspartate aminotransferase
CLDQ: Chronic liver disease questionnaire
C–P: Child–Pugh
ELF: Enhanced liver fibrosis
ET: Early termination
FDA: Food and Drug Administration
FXR: Farnesoid X receptor
GGT: γ-Glutamyl transferase
HCC: Hepatocellular carcinoma
HE: Hepatic encephalopathy
HRQOL: Health-related quality of life
IBD: Inflammatory bowel disease
IgG4: Immunoglobulin G4
INR: International normalized ratio
IPSCSG: International PSC Study Group
MELD: Model for end-stage liver disease
MRCP: Magnetic resonance cholangiopancreatography
PK: Pharmacokinetics
PSC: Primary sclerosing cholangitis
PSC-PRO: Primary sclerosing cholangitis-patient-reported outcome
OLE: Open-label extension
SIBDQ: Short inflammatory bowel disease queistionnaire
UDCA: Ursodeoxycholic acid
VCTE: Vibration-controlled transient elastography
